# Xylazine co-occurrence with illicit fentanyl is a growing threat in the Deep South: a retrospective study of decedent data

**DOI:** 10.1186/s12954-024-00959-2

**Published:** 2024-02-20

**Authors:** William Bradford, Mary Figgatt, Karen S. Scott, Stacy Marshall, Ellen F. Eaton, Daniel W. Dye

**Affiliations:** 1 Division of Infectious Diseases, University of Alabama at Birmingham, Boshell Diabetes Building 8th Floor 1808 7th Ave S, Birmingham, AL 35233 USA; 2 Division of Laboratory Medicine, University of Alabama at Birmingham, Birmingham, USA; 3 Department of Emergency Medicine, University of Alabama at Birmingham, Birmingham, USA; 4 Jefferson County Coroner/Medical Examiner’s Office, Birmingham, USA; 5 Department of Pathology, University of Alabama at Birmingham, Birmingham, USA

**Keywords:** Xylazine, Fentanyl, Opioid use disorder, Overdose, Opioid epidemic

## Abstract

**Background:**

Xylazine is a dangerous veterinary sedative found mainly in illicit fentanyl in the Northeast and Midwest. Its role in the Deep South overdose crisis is not well-characterized.

**Methods:**

We conducted a retrospective review of autopsy data in Jefferson County, Alabama to identify trends in xylazine prevalence among people who fatally overdosed from June 2019 through June 2023.

**Results:**

165 decedents met inclusion criteria. While the first identified xylazine-associated overdose was in June 2019, xylazine has become consistently prevalent since January 2021. All cases of xylazine-associated fatal overdoses were accompanied by fentanyl, and most (75.4%) involved poly-drug stimulant use. The average age was 42.2, and most decedents were white (58.8%) and male (68.5%). Overall, 18.2% of people were unhoused at the time of death.

**Discussion:**

Xylazine is prevalent in the Deep South. Efforts to promote harm reduction, publicly viewable drug supply trends, and legalization of drug checking and syringe service programs should be prioritized.

## Background

Since the early 2010s, the unregulated opioid supply in the U.S. has experienced major fluctuations in its chemical makeup, notably via the emergence of fentanyl [1]. Shortly after the start of the 2020 COVID-19 pandemic, a newer additive called xylazine became increasingly recognized in the unregulated opioid supply in parts of the United States [2]. Xylazine is a sedative that is used in veterinary medicine; however, reports of exposure in humans date back to the 1970s and include both direct use of the veterinary medicine, as well as unintentional use through additives in the opioid supply. Many of its acute health effects are consistent with other sedatives: heavy sedation, respiratory depression, hypotension, and bradycardia [3]. However, severe skin wounds have been increasingly linked to xylazine as a complication of use [4, 5], likely as a complication of more chronic exposure to xylazine and possibly direct tissue injury from extravasation [6]. Its role in contributing to or protecting against overdose death is currently unclear, with some data suggesting lower risks of fatal overdose [7] and others indicating the opposite (largely based on plausible synergy in promoting CNS depression between xylazine and fentanyl) [8]. High prevalence of xylazine in the opioid supply has been identified in many areas in the Northeast United States, first noted in Philadelphia. Other geographical regions where it has been increasingly identified include Maryland, Connecticut, and Wisconsin [2, 9].

The Deep South faces many intersecting barriers to respond to the influx of xylazine. These barriers include regressive policies that prevent public health approaches to overdose prevention, a lack of consistent toxicology testing and reporting, and limited harm reduction services to reach the community [10]. Xylazine has been identified in one of the most populated counties in the Deep South—Jefferson County, Alabama [2]. However, little is known about regional trends and populations impacted by xylazine in the area. The objective of this analysis was to examine monthly trends in the emergence of xylazine in the Deep South as well as the characteristics of people who experience a fatal overdose associated with xylazine in Jefferson County.

## Methods

The Jefferson County Coroner/Medical Examiner's Office (JCCMEO) performs postmortem toxicology testing of decedents whose deaths fall under the jurisdiction of the office. The office investigates all “accidental” deaths that occur in Jefferson County, Alabama which includes drug overdose deaths. Postmortem toxicology testing of suspected drug overdoses, in addition to a forensic autopsy, is performed in accordance with the National Association of Medical Examiners Forensic Autopsy Performance Standards. We evaluated all decedents with xylazine detected on toxicology testing through the medical examiner’s database from June 2019 (date of first detection) through June 2023. We excluded decedents from motor vehicle fatalities and homicides to capture only overdose fatalities. Overdose deaths were quantified from publicly reported data obtained by previously outlined methods [11].

For patients who met inclusion criteria, we abstracted information from the medical examiner autopsy records including sociodemographic information and data on all drugs detected from toxicology testing. The means of toxicology testing were enzyme multiplied immunoassay technique (EMIT) for screening, gas chromatography/mass spectrometry (GC/MS) for confirmatory analysis, and liquid chromatography tandem mass spectrometry (LC–MS/MS) for confirmatory analysis in a small number of cases where send out testing was required. Sex was assigned based on comprehensive autopsy findings. Race was assigned by Driver’s License, other formal identification-reported race, or by family report. Housing status was defined by two clinician medical review of investigative information in the database for each included death. In situations where it was not possible to determine with certainty whether a person was housed or unhoused, the designation “unable to determine” was assigned. We calculated descriptive statistics and summarized demographic and clinical factors using measures of central tendency (means and medians), dispersion (range and standard deviation), and distribution (frequency and percentage). There were no missing data on the variables of interest. All analyses were conducted using JMP software (v.17.0) and Microsoft Excel.

## Results

**Fig. 1:**
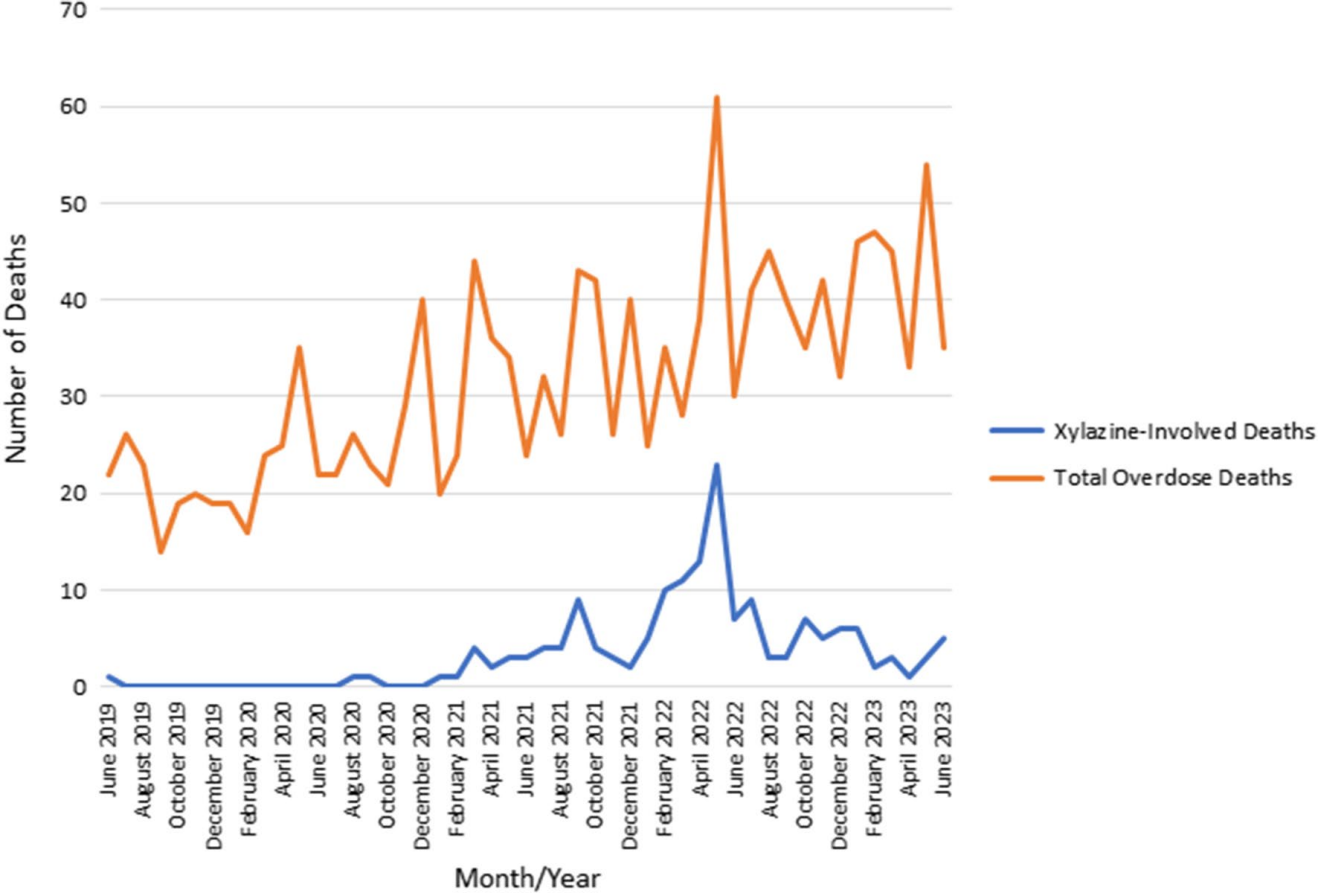
Total and xylazine-involved overdose deaths in Jefferson County, June 2019 through June 2023

**Fig. 2:**
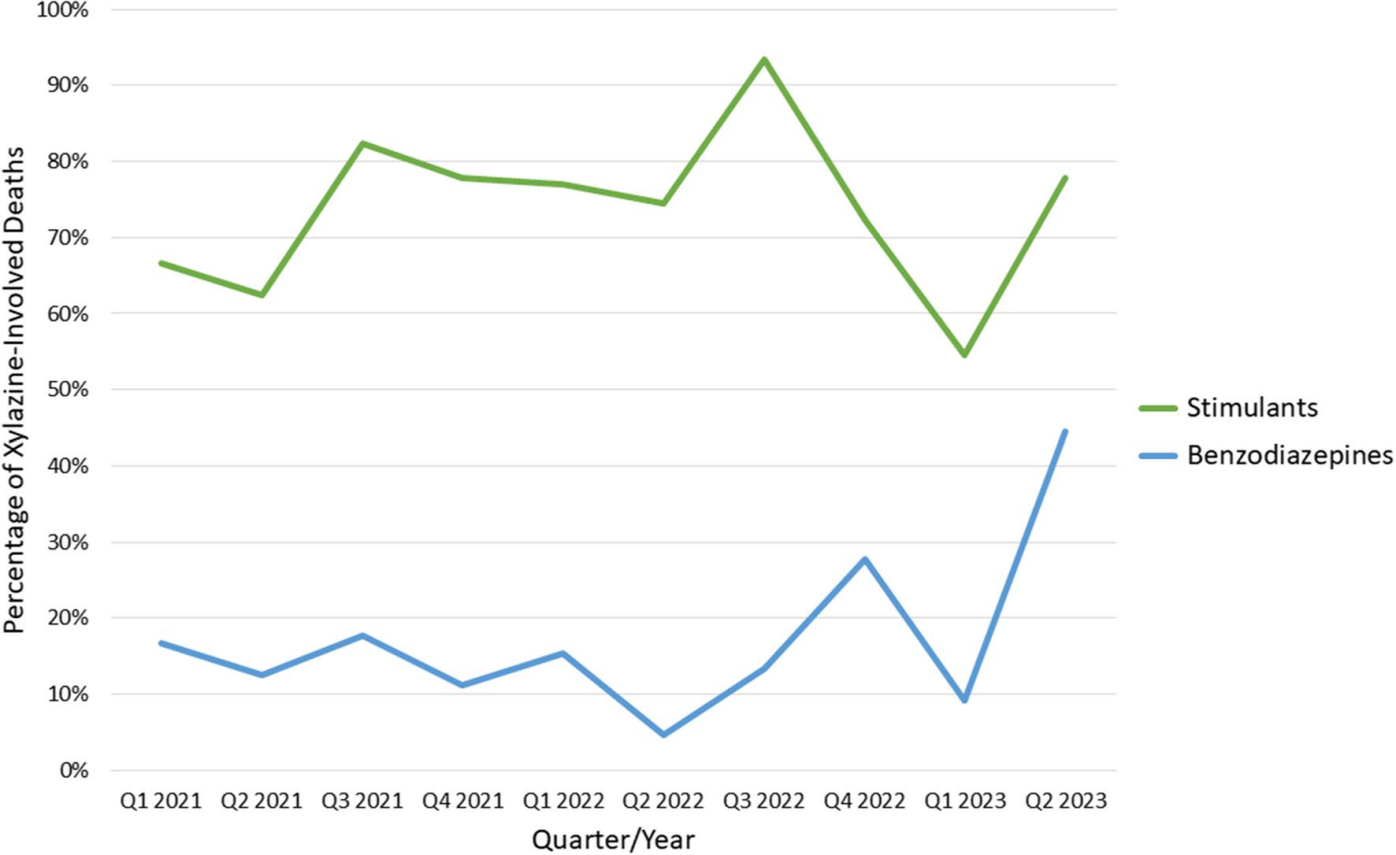
Percentage of stimulants and benzodiazepines in persons with fatal xylazine-associated deaths in Jefferson County, Q1 2021 through Q2 2023, of whom 100% had opioids on toxicology

Of the 165 decedents who died from overdose and had xylazine detections, the average age was 42.2 years, and most decedents (*n* = 113, 68.5%) were male sex (see Table 1). Most were white (*n* = 97, 58.8%), followed by black (*n* = 62, 37.6%) and Hispanic (*n* = 6, 3.6%). The majority of decedents were housed (*n* = 133, 80.6%), with 30 (18.2%) unhoused and 2 (1.2%) “unable to determine.” Xylazine was detected in waves (see Fig. 1), with the largest wave having a peak in March 2022. Xylazine was associated with 39.3% of total overdose deaths during the study period, and high xylazine prevalence occurring during a large spike in total overdose deaths. Figure 2 lists other major substance classes that were detected in xylazine overdose deaths during the period of persistent xylazine prevalence from January 2021 through June of 2023. Fentanyl was detected in all 165 (100%) deaths where xylazine was also present, while stimulants were detected in 124 (75.2%) and benzodiazepines in 25 (15.2%). The most commonly detected stimulant was cocaine (75, 45.7%) followed by methamphetamine (59, 35.8%) and amphetamine (47, 28.5%). Amphetamine was detected without the presence of methamphetamine in six (3.6%) cases. Fentanyl analogs were rarely seen, with fluorofentanyl seen in only 4 (2.4%) deaths. Levamisole was detected in 3 (1.8%) deaths.

**Table 1:** Demographic and clinical characteristics of overdose decedents with xylazine detected during 2019–2023 in Jefferson County, Alabama (*N* = 165)

Characteristic	*N* (%)
Age	
Mean ± SD	42.2 ± 11.7
Median, min to max	42, 4 to 70
Sex	
Female	52 (31.5)
Male	113 (68.5)
Race	
White	97 (58.8)
Black	62 (37.6)
Hispanic	6 (3.6)
Housing status	
Housed	133 (80.6)
Unhoused	30 (18.2)
Unable to determine	2 (1.2)
Year identified	
2019	1 (0.6)
2020	2 (1.2)
2021	40 (24.2)
2022	102 (61.8)
2023 (through June)	20 (12.1)
Drug overlap (broad category)	
Fentanyl	165 (100)
Stimulant	124 (75.2)
Benzodiazepine	25 (15.2)

*Max* maximum, *Min* minimum, *SD* standard deviation

## Discussion

Xylazine has been persistently present in one Deep South county, first arriving in 2019 and becoming consistently prevalent since January 2021. This is concordant with increasing prevalence elsewhere including in the Midwest and along the East Coast [2]. Fentanyl was co-detected in 100% of deaths, consistent with current understanding that xylazine is used mainly as a “cutting” agent in illicit fentanyl preparations. This also reinforces the prevailing theory that fentanyl, not xylazine itself, is the main driver of overdose death [12].

After fentanyl, stimulants were the next most common drug category associated with fatal overdose. Amphetamine was frequently detected, likely reflecting its status as a metabolite of methamphetamine. In the minority of cases where amphetamine was detected alone, it was most likely being used to treat attention deficit disorders rather than as a substance of abuse. Benzodiazepines and other drug classes were rarely detected. Sociodemographic characteristics of xylazine overdose victims were similar to those of Jefferson County’s publicly reported drug overdose deaths during the 2020–2022 timeframe, during which time the typical overdose victim was similarly male (69.6% of all overdose victims), white (55.5% of all overdose victims), and middle aged (most overdose deaths occurred in the 40–49 year old age group) [13]. This suggests that similar groups of persons were using both xylazine-containing substances and non-xylazine-containing substances.

Unlike larger northeastern and midwestern municipalities with well-developed drug surveillance and public health infrastructure, many jurisdictions in the Deep South lack a robust public health response to this growing threat, with proximate barriers including a lack of access to legal syringe service programs (SSP’s) and other harm reduction supplies like xylazine test strips [10]. More globally, high levels of stigma, limited avenues for dissemination of harm reduction techniques, poor data collection practices, limited surveillance of emerging substances with overdose potential, and heavy criminalization continue to represent major barriers to harm reduction practice implementation in nearby rural communities [14].

Significantly, many Deep South states lack an apparatus for comprehensive death evaluation, which may be leading to significant undercounting of both the number of overdose deaths along with poor detection of xylazine and other novel adulterants (e.g., medetomidine). Most death investigations in rural jurisdictions are conducted by a Coroner, an elected lay official who has limited training in death examination compared to Medical Examiners (ME’s), who are most often appointed pathologists (or forensic pathologists) with additional training in medicolegal death investigations. The accuracy of reporting of drug related fatalities depends largely on the Medicolegal Death Investigation (MDI) system that covers the region. The training and experience of coroners vary greatly throughout the United States [15]. A review of information from deaths that occurred in 2014 and in 2018 showed that coroner systems were associated with a greater likelihood of not indicating what drug (or drugs) caused an overdose death. Furthermore, this was statistically significant when comparing rural and urban counties [16]. Coroner systems had a lower likelihood of performing an autopsy in suspected drug related deaths. In addition, a coroner system is associated with a lower likelihood of recording opioid-related deaths. These data indicate that accurate reporting of overdose deaths depends greatly on the MDI system [15]. In Alabama, coroners oversee death investigations in all counties except Jefferson County, Alabama and most death investigations in the state occur in Coroner jurisdictions.

There were several limitations to this study: most notably, only cases referred to the ME were evaluated. Approximately 19% of deaths in Jefferson County are referred for formal death examination, so the prevalence of xylazine-associated overdose may be higher, especially in certain populations and regions that may be less likely to receive a comprehensive formal death investigation like ethnic minorities and males [15]. Additionally, this is a single county experience. However, Jefferson County is the most populous county in Alabama, containing some 13% of the total state population by 2020 census numbers, but an outsize 26% of the state’s reported overdose fatalities [17]. Our study is strengthened by the routine use of a high-quality detection method (i.e., mass spectrographic methods) that were capable of identifying xylazine, through NIST library searching, even before routine surveillance for the compound was nationally available. Thus, the first appearance of the compound can be inferred with high certainty.

We recommend several steps to better prepare Deep South states for the xylazine/fentanyl syndemic. First and foremost, immediate legalization of xylazine test strips should be pursued in jurisdictions where they are currently illegal. People who use drugs (PWUD) generally prefer to avoid xylazine-containing fentanyl [18] when able, and empowering them to do so with a tool similar to fentanyl test strips could be a powerful tool in reducing harm from xylazine. Second, more consistent testing is needed for xylazine and other concerning contaminants, and results must be conveyed to both communities and providers to inform educational initiatives and an appropriate public health response. Recent efforts to expand drug checking improve awareness of PWUD and empower them to be vigilant for substances like xylazine [8, 9]. Implementation of these services in the Deep South is necessary. Next, the use of coroners rather than medical examiners in many parts of the Deep South including Louisiana, Alabama, South Carolina, and Georgia, limits the ability to detect trends in overdose death in general, especially emerging drug trends like xylazine overdose. More standardized protocols and funding to pursue toxicology testing in rural coroner systems may yield more accurate estimates of xylazine- and opioid-related overdose death. Finally, educational initiatives should be undertaken directed at both medical providers and PWUD to educate them on the best harm reduction practices associated with xylazine. Early recognition of xylazine-related wounds, in particular, could potentially prevent the development of limb threatening soft tissue destruction. In addition to improved clinic-focused interventions to improve xylazine wound recognition and treatment currently underway, coroners and ME’s should be aware of the characteristic features of these wounds as well so that they can request appropriate toxicologic testing and contribute to community awareness of this important topic.

Xylazine is a rapidly emerging and serious public health threat that merits aggressive intervention and continued close monitoring [19]. It has been prevalent in one Deep South county for at least 3 years and may be more widespread given resource availability in different MDI systems across the region. Future research should focus on establishing the role of xylazine adulteration in promoting clinical diseases outside of soft tissue infection and improving implementation of best harm reduction practices in order to empower PWUD to avoid this dangerous adulterant.

## Data Availability

The datasets used and/or analyzed during the current study are available from the corresponding author on reasonable request.

## Abbreviations

GC/MS: Gas chromatography/mass spectrometry
LC–MS/MS: Liquid chromatography/mass spectrometry
JCCMEO: Jefferson County Coroner/Medical Examiner's Office
MDI: Medicolegal Death Investigation
ME: Medical examiner
PWUD: People who use drugs
SSP: Syringe service program

## References

[1] Kariisa M, O'Donnell J, Kumar S, Mattson CL, Goldberger BA (2023). Illicitly manufactured fentanyl-involved overdose deaths with detected xylazine—United States, January 2019-June 2022. Morb Mortal Wkly Rep.

[2] Friedman J, Montero F, Bourgois P, Wahbi R, Dye D, Goodman-Meza D (2022). Xylazine spreads across the US: a growing component of the increasingly synthetic and polysubstance overdose crisis. Drug Alcohol Depend.

[3] Zagorski CM, Hosey RA, Moraff C, Ferguson A, Figgatt M, Aronowitz S (2023). Reducing the harms of xylazine: clinical approaches, research deficits, and public health context. Harm Reduct J.

[4] Ball NS, Knable BM, Relich TA, Smathers AN, Gionfriddo MR, Nemecek BD (2022). Xylazine poisoning: a systematic review. Clin Toxicol (Phila).

[5] D'Orazio J, Nelson L, Perrone J, Wightman R, Haroz R (2023). Xylazine adulteration of the heroin-fentanyl drug supply: a narrative review. Ann Intern Med.

[6] Malayala SV, Papudesi BN, Bobb R, Wimbush A (2022). Xylazine-induced skin ulcers in a person who injects drugs in Philadelphia, Pennsylvania, USA. Cureus.

[7] McFadden R, Wallace-Keeshen S, Petrillo Straub K, Hosey RA, Neuschatz R, McNulty K (2024). Xylazine-associated wounds: clinical experience from a low-barrier wound care clinic in Philadelphia. J Addict Med.

[8] Nunez J, DeJoseph ME, Gill JR (2021). Xylazine, a veterinary tranquilizer, detected in 42 accidental fentanyl intoxication deaths. Am J Forensic Med Pathol.

[9] Zhu DT (2023). Public health impact and harm reduction implications of xylazine-involved overdoses: a narrative review. Harm Reduct J.

[10] Fernández-Viña MH, Prood NE, Herpolsheimer A, Waimberg J, Burris S (2020). State laws governing syringe services programs and participant syringe possession, 2014–2019. Public Health Rep.

[11] Cain MD, McGwin G, Atherton D (2016). Surveillance of drug overdoses using google fusion tables. Acad Forensic Pathol.

[12] Love JS, Levine M, Aldy K, Brent J, Krotulski AJ, Logan BK (2023). Opioid overdoses involving xylazine in emergency department patients: a multicenter study. Clin Toxicol (Phila).

[13] Office JCCMEs. Annual Report of the Jefferson County Coroner/Medical Examiner's Office. Birmingham, AL; 2020–2022.

[14] Childs E, Biello KB, Valente PK, Salhaney P, Biancarelli DL, Olson J (2021). Implementing harm reduction in non-urban communities affected by opioids and polysubstance use: a qualitative study exploring challenges and mitigating strategies. Int J Drug Policy.

[15] Boslett AJ, Denham A, Hill EL, Adams MCB (2019). Unclassified drug overdose deaths in the opioid crisis: emerging patterns of inequity. J Am Med Inform Assoc.

[16] Denham A, Vasu T, Avendano P, Boslett A, Mendoza M, Hill EL (2022). Coroner county systems are associated with a higher likelihood of unclassified drug overdoses compared to medical examiner county systems. Am J Drug Alcohol Abuse.

[17] County-level provisional drug overdose death counts. National Center for Health Statistics. 2023.

[18] Reed MK, Imperato NS, Bowles JM, Salcedo VJ, Guth A, Rising KL (2022). Perspectives of people in Philadelphia who use fentanyl/heroin adulterated with the animal tranquilizer xylazine; Making a case for xylazine test strips. Drug Alcohol Depend Rep.

[19] Gupta R, Holtgrave DR, Ashburn MA (2023). Xylazine—medical and public health imperatives. N Engl J Med.

